# Application of a Carbon Fiber Microelectrode as a Sensor for Apocynin Electroanalysis

**DOI:** 10.3390/ma17071593

**Published:** 2024-03-30

**Authors:** Slawomir Michalkiewicz, Agata Skorupa, Magdalena Jakubczyk, Karolina Bębacz

**Affiliations:** 1 Institute of Chemistry, Jan Kochanowski University, PL-25406 Kielce, Poland; agata.skorupa@ujk.edu.pl (A.S.); magdalena.jakubczyk@ujk.edu.pl (M.J.); 2 Central Office of Measures, PL-00139 Warsaw, Poland; karolina.bebacz@gum.gov.pl

**Keywords:** apocynin, carbon fiber microelectrode, electrochemical properties, determination, voltammetry, acetic acid, acetonitrile, herbal extracts, dietary supplements

## Abstract

In this study, a carbon fiber microelectrode (CF) was applied for the investigation of the electrochemical behavior of the natural antioxidant, apocynin (APO). Given the limited solubility of APO in water, a mixture of anhydrous acetic acid (AcH) with 20%, *v*/*v* acetonitrile (AN) and 0.1 mol L^−1^ sodium acetate (AcNa) was used. The electrochemical properties of APO were examined through linear sweep voltammetry (LSV), differential pulse voltammetry (DPV), and cyclic voltammetry (CV). The anodic oxidation of APO, which is the basis of the method used, proved to be diffusion-controlled and proceeded with a two-electron and one proton exchange. Both radicals and radical cations, arising from the first and second step of electrode reactions, respectively, underwent subsequent chemical transformations to yield more stable final products (*E*_q_*C*_i_*E*_i_*C*_i_ mechanism). Using optimized DPV conditions, the anodic peak current of APO at a potential of 0.925 V vs. Ag/AgCl showed a good linear response within the concentration range of 2.7 × 10^−6^–2.6 × 10^−4^ mol L^−1^. The detection and quantification limits were determined as 8.9 × 10^−7^ and 2.7 × 10^−6^ mol L^−1^, respectively. The developed DPV method enabled the successful determination of APO in herbal extracts and in dietary supplements. It should be noted that this is the first method to be used for voltammetric determination of APO.

## 1. Introduction

**Figure 1:**
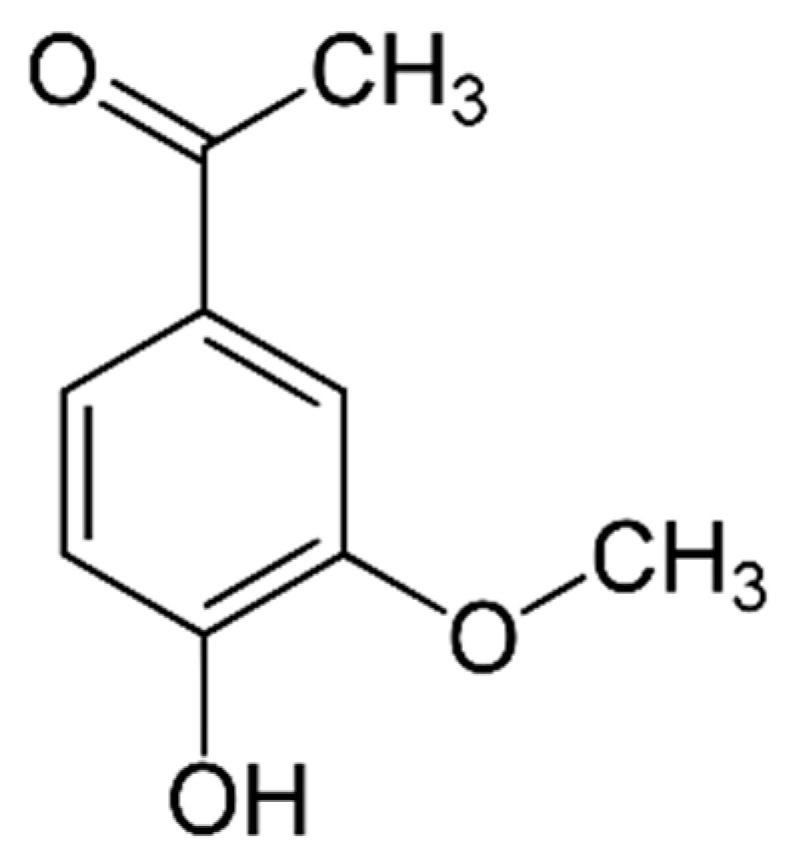
Molecular structure of apocynin.

Apocynin (APO, 4-hydroxy-3-metoxyacetophenone, C_9_H_10_O_3_; Figure 1) is an interesting, naturally small molecule with antioxidant properties, classified as an acetophenone. Structurally, it resembles vanillin, hence it is often called acevanillione [1].

APO was isolated from the roots of the Canadian plant *Apocynum cannabinum*, from which it took its common name. It was also identified in the roots of another plant, *Picrorhiza kurroa* (Kutki) growing in the Himalayas [1,2,3,4,5,6]. The extract of this herb has long been used in India and Sri Lanka in traditional Indian medicine (Ayurveda). It has been used to treat swelling, fever, arthritis, and symptoms of various ailments, such as asthma, jaundice, heart problems, and liver dysfunction [1,2,3,5,6,7,8,9,10,11,12]. Therefore, there is an increasing interest in the medicinal properties of apocynin and its potential use in pharmaceutical preparations and in dietary supplements. Extracts containing this substance have already been appreciated in the USA, where they are used to treat liver diseases and other ailments related to oxidative stress [7]. Many studies to date have confirmed the healing properties of apocynin [1,2,5,9,10,11].

Animal studies have shown that APO is an antioxidant that inhibits reactive oxygen species (*ROS*) in the liver, heart, and brain. This ability is due to its role as an inhibitor of NADPH oxidase. Consequently, apocynin exhibits strong anti-inflammatory properties. The antioxidant properties of APO determine its potential use in treating ailments caused by oxidative stress, mainly in cardiovascular, respiratory, and neurodegenerative diseases, such as Alzheimer’s and Parkinson’s diseases [2,5,7,8,9]. Additionally, the use of APO as a drug can be attributed to its low toxicity, high efficacy, and stability. Studies have shown that APO protects against stroke and its effects [2,3]. The substance is also promising for the treatment of hypertension and atherosclerosis [2,13]. As a natural compound, APO may provide an alternative to steroidal and non-steroidal dermatological drugs, which cause a number of side effects [1]. The potential of apocynin as a complementary drug in the treatment of COVID-19 has also been demonstrated [13].

The physicochemical and pharmacological properties of apocynin have been the subject of many articles to date [1,2,3,4,5,6,8,9,10,11]. In contrast, there are only a few publications on its identification and determination in real samples [14,15,16,17,18]. For this purpose, we used high-performance liquid chromatography with UV detection (HPLC-UV) [14,15] and liquid chromatography with mass spectrometry (LC-MS) [17,18] or tandem mass spectrometry (LC-MS/MS) [16]. Chromatographic methods are extremely effective in achieving low detection and quantification limits. However, they have several disadvantages, including expensive instrumentation and long and complicated sample pre-treatment. Electroanalytical methods offer a good alternative with a fast and cost-effective on-site analysis, high specificity, and sensitivity. They are based on the electrochemical properties of analytes. These properties have not yet been studied in detail. The literature does not provide any information on the possibility of its voltammetric determination. To the best of our knowledge, there is only one paper regarding the electrochemical behavior of apocynin [10]. The oxidation potential of APO was only determined and compared with another compound having antioxidant properties, protocatechuic acid (3,4-dihydroxybenzoic acid), which naturally occurs in green tea. The study was carried out using cyclic voltammetry (CV) on a glassy carbon working electrode (GCE) in 0.2 mol L^−1^ phosphate buffer with pH 7. The oxidation peak potential for APO was 0.76 V vs. Ag/AgCl. Its relatively low value confirms that this substance has antioxidant properties and the ability to scavenge free radicals [10].

To date, no information has been made available regarding the electrochemical behavior of APO in acetic acid (AcH). Moreover, voltammetry has not yet been applied for the quantification of this substance, both in aqueous and in organic solvent environments. This solvent has many beneficial properties, including a relatively wide potential window [19]. It is also safe for humans and the environment. The low electrical conductivity of acetic acid leads to uncontrolled ohmic potential drops (*IR*) between the electrodes. To eliminate this phenomenon, microelectrodes (e.g., made of carbon fiber, CF) are used in voltammetric measurements.

The aim of this work was to investigate the electrochemical properties and to develop a simple, fast, and highly sensitive voltammetric procedure for the determination of APO in herbal extracts and dietary supplement samples using a carbon fiber microelectrode (CF) in acetic acid solution. Importantly, this is the first voltammetric method employed to determine apocynin, and it is based on a comprehensive analysis of apocynin’s electrochemical properties. 

## 2. Materials and Methods

### 2.1. Chemicals

The following chemicals were used in the experiments: apocynin (APO) 98%; sodium perchlorate; NaClO_4_, anhydrous, p.a. (both Sigma-Aldrich, Saint Louis, MO, USA) and sodium acetate; and CH_3_COONa (AcNa), anhydrous > 99.0% (Fluka, Charlotte, NC, USA). All solutions were prepared with Merck solvents: anhydrous acetic acid, CH_3_COOH (AcH), p.a., ACS and acetonitrile, and CH_3_CN (AN), p.a. Quantitative studies were carried out using the Kutki dietary supplement (Aurospirul, Auroville, India). Rosemary samples in dry form (Kamis S.A., Stefanowo, Poland), enriched with a known amount of APO, constituted the matrix in the recovery studies of analyte in extracts.

### 2.2. Apparatus

Voltammetric experiments were conducted using a Model M161E electrochemical analyzer connected with a Model M162 preamplifier and controlled using mEALab Version 2.1 software (mtm-anko, Krakow, Poland). The software was also equipped with a program for analytical determination by the standard addition method. All experiments were carried out using a three-electrode cell. Disk microelectrodes made of Pt, Pd, Au (all 25 μm in diameter, Mineral, Warsaw, Poland) and carbon fiber, CF with a diameter of 33 μm (BASi, West Lafayette, IN, USA) or glassy carbon disk, and GC (3 mm in diameter, BASi, West Lafayette, IN, USA) were used as working electrodes. Platinum wire and Ag/AgCl containing 3 mol L^−1^ KCl (both Mineral, Warsaw, Poland) were used as auxiliary and reference electrodes, respectively. To avoid leakage of water and chloride ions into the solutions tested, the reference electrode was isolated by a salt bridge with a frit of Vicor-Glass filled with a supporting electrolyte. Before each series of experiments, the surface of the working electrode was polished using 0.05 μm alumina, rinsed with water, and dried. To minimize electrical interferences during the microelectrode experiments, the electrochemical cell was enclosed in a grounded Faraday cage.

The Model inoLab 720 conductivity meter equipped with a conductivity cell TetraCon 325 (WTW, Weilheim, Germany) was used to measure the specific conductivity of the solutions. The cell constant, *k*, was determined to be 0.475 cm^−1^. 

The CX-732 multifunctional computer meter equipped with a pH sensor, comprising a glass indicator electrode and Ag/AgCl reference electrode (Elmetron, Zabrze, Poland), was used for pH measurements.

Conductivity and pH measurements were carried out with automatic temperature compensation up to 25 °C, while the voltammetric experiments were carried out at room temperature 25 ± 1 °C.

The Yellow Line OS 5 orbital laboratory shaker (IKA Werke, Weilheim, Germany) was used to extract the analyte from the spiked herbal samples.

### 2.3. Electrochemical Procedures

The electrochemical behavior of APO in optimized conditions was studied in acetic acid containing acetonitrile (20%, *v*/*v*) and 0.1 mol L^−1^ sodium acetate on a CF disk microelectrode with a diameter of 33 μm. For this purpose, linear sweep (LSV) and differential pulse voltammetry (DPV) were applied. To ensure steady-state conditions, the LSV curves were recorded at a slow scan rate of 6.2 mV s^−1^. To obtain the best-shaped DPV curves and satisfactory sensitivity of the determinations, optimized parameters were used: amplitude, d*E* of 30 mV, potential step, *E*_s_ of 5 mV, and pulse width of 60 ms. The voltammograms for both LSV and DPV were recorded within the potential range from −0.6 to 1.7 V vs. Ag/AgCl. Some voltammetric investigations were performed using cyclic voltammetry (CV) on a GC electrode. The solutions tested then contained the concentration of the supporting electrolyte increased to 0.5 mol L^−1^. CV curves were recorded for a scan rate range of 6.2 to 2000 mV s^−1^.

The DPV technique was used to determine the analytical parameters of the developed method and for voltammetric determinations. The test solutions contained APO at a concentration ranging from 6.0 × 10^−7^ to 7.2 × 10^−4^ mol L^−1^. APO signals were measured after the baseline correction.

Control determinations and real sample analysis were conducted using the standard addition method. The procedure for APO determination in the dietary supplement and in spiked herbal samples involved extraction with acetonitrile (10 mL/1 g herbal) and shaking for the optimized time of 7 h. The extracts were next filtered through a 0.45 μm membrane filter. The solutions tested were prepared in a 25 mL volumetric flask by diluting a known volume of extracts in acetic acid and adding AN and AcNa in the amounts, allowing the concentrations to remain constant at 20% (*v*/*v*) and 0.1 mol L^−1^, respectively. The concentration of the apocynin standard solution was depended on the analyte amount present in the test solutions. DPV voltammograms were recorded for a sample volume of 2.0 mL and after four consecutive additions of the APO standard in portions of 50 µL. The determinations were repeated five times.

## 3. Results and Discussion

### 3.1. Study of the Best Voltammetric Conditions

**Figure 2:**
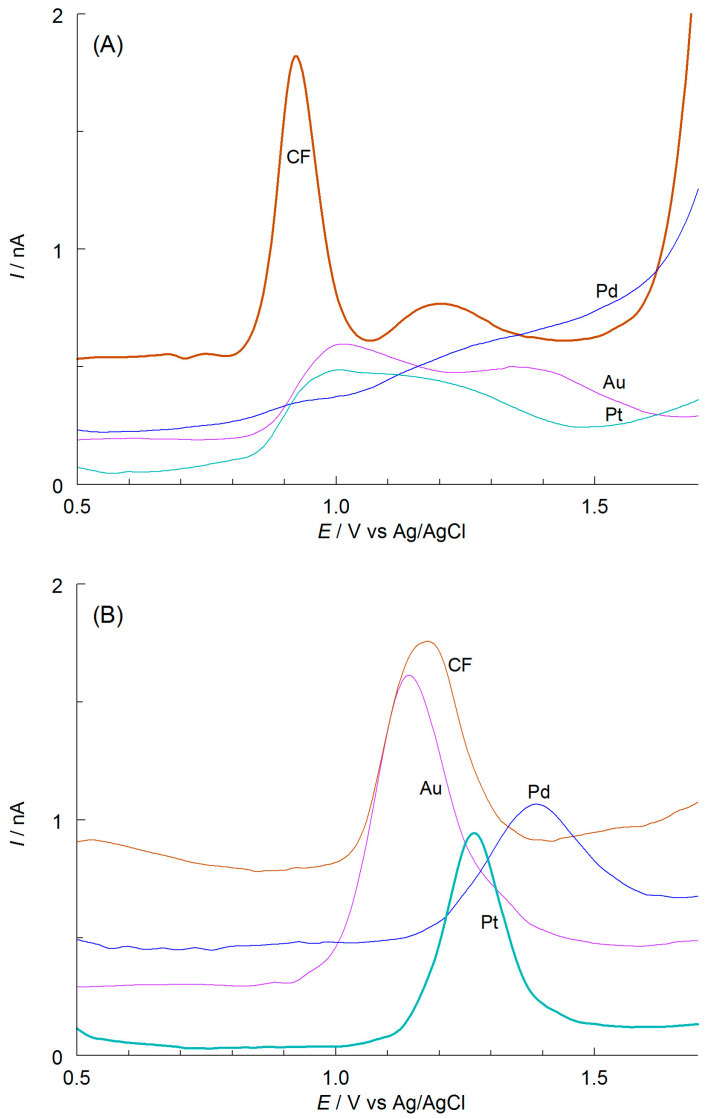
DPV curves of 0.50 mmol L^−1^ APO recorded in AcH with 20% AN (*v*/*v*) and 0.1 mol L^−1^ (**A**) CH_3_COONa or (**B**) NaClO_4_. The microelectrode disk material is given near the curves.

The first stage of the voltammetric experiments was to select the optimal solution composition and working electrode material. Given the limited solubility of APO in aqueous solutions (only in hot water [20]), anhydrous acetic acid was used as the environment. AcH is able to dissolve both the hydrophobic analytes with their matrices and the hydrophilic supporting electrolyte necessary for voltammetric studies. However, its low electrical permittivity (ε = 6.2 [21]) may lead to an unfavorable ohmic potential drop, *IR* between the electrodes. This is caused by slight dissociation of the supporting electrolyte in this organic solvent. To prevent that, AN (ε = 35.9 [21]) was added to the acetic acid at 20%, *v*/*v*. Its presence increased the specific conductivity of the mixed solvent (from 0.08 to 1 μS cm^−1^), thus facilitating charge transfer between the electrodes. For the same reason, microelectrodes were used in the study. Small currents generated on such working electrodes (nA or pA) make the *IR* drop negligible, even with significant solution resistances. When selecting the supporting electrolyte, two popular salts with different acid-based properties were considered: sodium acetate and sodium perchlorate (basic and acidic properties, respectively). Their concentration in the tested solutions was 0.1 mol L^−1^. The specific conductivities of the solutions containing CH_3_COONa and NaClO_4_ (383 and 1358 μS cm^−1^, respectively) increased significantly compared to the mixed solvent. The effect of the *IR* drop on the voltammetric results for this medium is insignificant. The DPV curves were initially recorded on the microelectrodes made of Pd, Au, Pt (all 25 μm in diameter), and CF (33 μm in diameter). The results are shown in Figure 2A,B. The best-shaped and symmetrical DPV curves were recorded on CF and Pt microelectrodes when the solutions contained sodium acetate and sodium perchlorate, respectively. AcNa was selected as the supporting electrolyte and CF as a working microelectrode material for future studies due to the improved reproducibility of successively recorded curves, lower peak potential of APO oxidation, *E*_p_ (0.925 vs. 1.265 V), smaller half-width, *W*_1/2_ (0.090 vs. 0.135 V), and higher peak current. This ensures high precision and sensitivity for APO determination, as well as optimal signal resolution.

### 3.2. Voltammetric Behavior of Apocynin in Acetic Acid Environment

**Figure 3:**
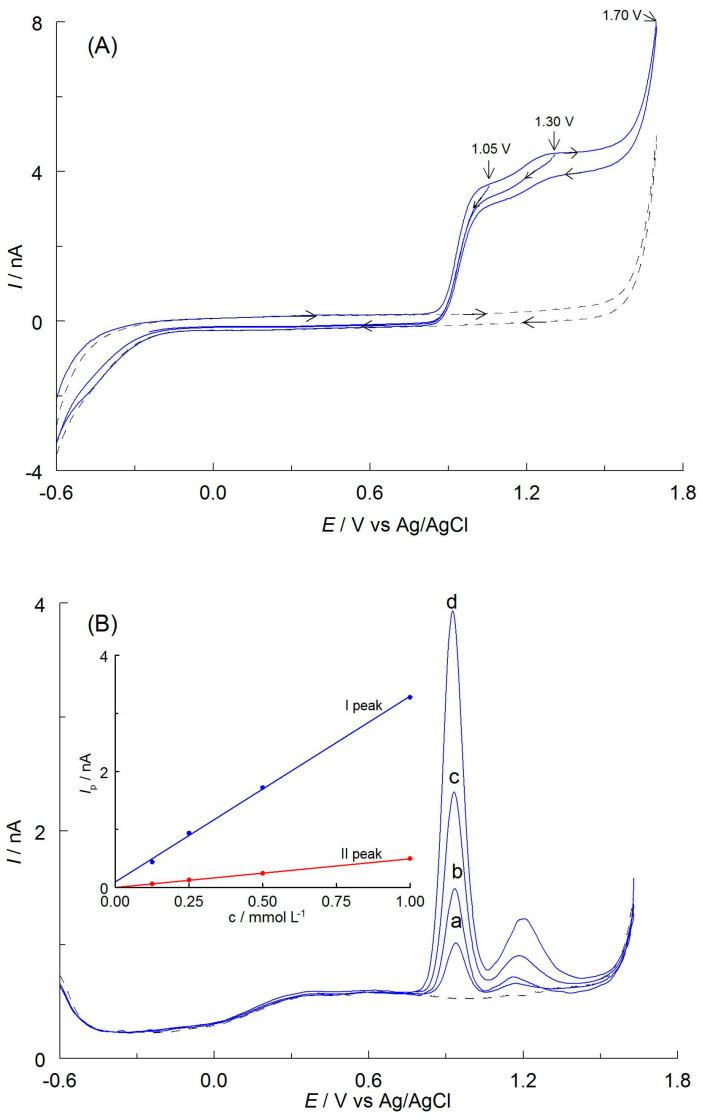
(**A**) LSV voltammograms (a scan rate of 6.2 mV s^−1^) of 0.50 mmol L^−1^APO recorded on CF microelectrode in AcH with 20% AN (*v*/*v*), 0.1 mol L^−1^ AcNa with different switching potentials, *E*_λ_ (given near the curves). The dashed line is the residual current. (**B**) DPV curves recorded in solutions containing APO with concentration of (a) 0.125, (b) 0.25, (c) 0.50, and (d) 1.0 mmol L^−1^. Inset: Calibration plot for DPV peak I and II.

The electrochemical properties of APO were investigated in an experimentally selected environment. The study began by using the linear sweep voltammetry technique (LSV) on the CF disk microelectrode. Under such conditions, a very well-shaped two-step anodic wave was obtained (Figure 3A). APO oxidation proceeds at a potential above 0.9 V. The unlimited current increase above 1.5 V is probably related to the oxidation of acetate ions (Kolbe reaction). After changing the polarization direction to cathodic, a slight hysteresis was observed. The size of the hysteresis decreased as the switching potential, *E*_λ_ decreased. The existence of the hysteresis may be attributed to the adsorption of APO oxidation reaction products and the resulting small changes on the electrode surface. Additionally, the presence of two waves in the LSV curves suggests a two-step APO oxidation process. This is also confirmed by the course of the DPV curves (Figure 3B) and the linear dependence of the oxidation current on the APO concentration for both signals observed in the LSV and DPV curves (inset on Figure 3B). The Tomes criterion was used to verify the reversibility of an electron exchange reaction. For a reversible process, the potential difference corresponding to 3/4 and 1/4 of the steady-state current for LSV curves (*E*_3/4_–*E*_1/4_) depends on the number of electrons exchanged, *n*. At 25 °C, this value should be 0.0564/*n* V [22,23]. The values obtained for both steps of the electrode reaction are 0.055 and 0.086 V, respectively. This suggests that the first step of anodic oxidation is nearly reversible and involves the exchange of one electron. The exchange of the second electron takes place irreversibly. The same conclusions can be drawn from an analysis of the peak width at half height, *W*_1/2_ of DPV curves. The theoretical value of this parameter for the exchange of one electron should be 0.0904 V at 25 °C [24]. The values obtained experimentally for the first and second steps of APO oxidation are 0.090 and 0.0150 V, respectively. They can be attributed to the reversible and irreversible exchange of one electron.

**Figure 4:**
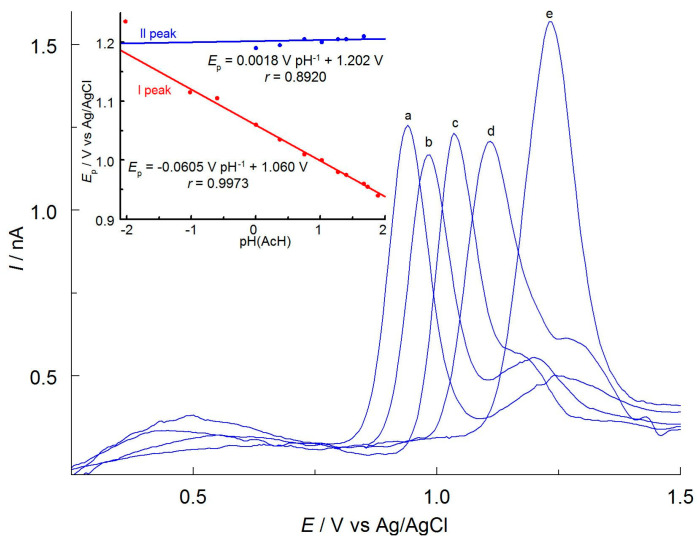
DPV curves of 0.50 mmol L^−1^ APO recorded on a CF microelectrode in AcH containing 20% AN (*v*/*v*) and different amounts of CH_3_COONa and NaClO_4_ (in mol L^−1^): (a) 0.10 and 0.00, (b) 0.05 and 0.05, (c) 0.012 and 0.088, (d) 0.001 and 0.099, (e) 0.00 and 0.10. Inset: Relationship between peak potentials I and II and pH (AcH).

To investigate the contribution of protons to the electrode reaction, the effect of pH on the DPV curves’ peak potential was examined. Relative pH changes were obtained by altering the concentration of sodium acetate. Acetate anions are the strongest base in anhydrous acetic acid. Therefore, their increasing concentration increases the pH of the solution. To keep the ionic strength constant, the solutions also contained varying amounts of NaClO_4_. The total concentration of the supporting electrolyte was always 0.10 mol L^−1^. The abbreviation pH (AcH) was used for the relative changes in pH values measured in an acetic acid medium. Figure 4 shows that an increase in concentration of acetate ions and pH (AcH) causes the first peak, *E*_p_, to shift toward less-positive potentials. The decrease in *E*_p_ with an increase in acetic anion concentration is due to the binding of protons, which are products of the first step of APO anodic oxidation. This shifts the equilibrium of the electrode reaction toward the products and thus facilitates the process. The position of the second signal is practically unchanged. Figure 4 inset shows a linear relationship between the potential of the first peak of APO oxidation on pH (AcH), with a slope of 0.0605 V pH^−1^. The value obtained is close to the expected theoretical value of 0.059 V pH^−1^ and indicates that the number of hydrogen ions and electrons involved in the first step of electrode reaction is equal. The potential of the second peak is practically independent of pH (AcH), which means that protons are not involved in the second step of APO anodic oxidation.

The results obtained suggest that the first step of the anodic oxidation of apocynin proceed with a quasireversible exchange of one electron and one proton. In contrast, the second part of the process can be attributed to an irreversible exchange of the second electron. The anodic currents corresponding to the second step of the electrode reaction (Figure 3) are significantly lower, indicating the instability of the product formed in the first step of APO oxidation. It probably undergoes a subsequent homogeneous reaction near the electrode surface, reducing the substrate concentration for the second step of electron exchange.

**Figure 5:**
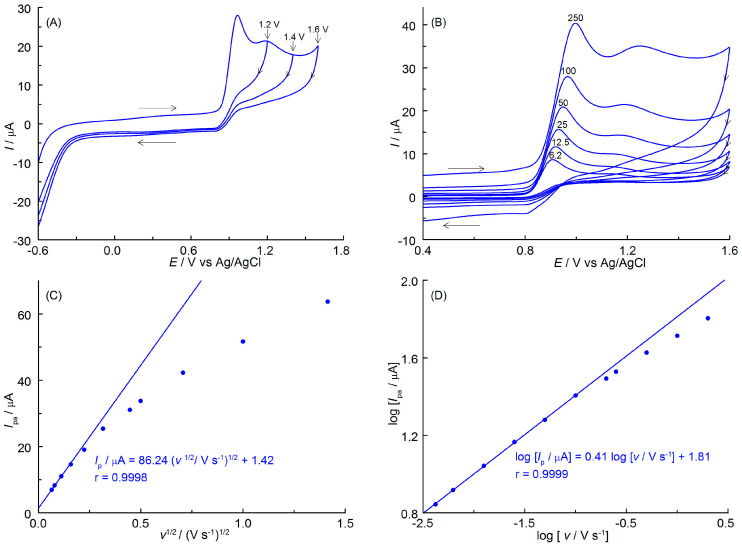
(**A**) Cyclic voltammograms (a scan rate of 100 mV s^−1^) of 1.0 mmol L^−1^ APO recorded on a GCE in AcH containing 20% AN (*v*/*v*) and 0.5 mol L^−1^ AcNa with different switching potentials *E*_λ_ (given near the curves). (**B**) CVs recorded in the scan rate range of 6.2–250 mV s^−1^. (**C**) The relationship between anodic peak current (*I*_pa_) and the square root of the scan rate (*v*^1/2^). (**D**) The dependence of logarithm of *I*_pa_ vs. logarithm of *v*.

The study then used cyclic voltammetry (CV) on a glassy carbon disk electrode to examine the behavior of APO, in particular, its oxidation products. To minimize the impact of a possible *IR* drop on the CV curves caused by the larger diameter of the working electrode and, consequently, higher currents, the concentration of the supporting electrolyte was raised from 0.1 to 0.5 mol L^−1^. The specific conductivity of these solutions increased to 1109 μS cm^−1^. A representative CV curve recorded at a scan rate of 100 mV s^−1^ is shown in Figure 5A. As can be seen, a well-defined main oxidation peak and a slightly shaped, broad second peak occur at potentials of about 0.95 and 1.2 V, respectively. The shape of the CV curve corresponds to the one recorded on the CF disk microelectrode and confirms the two-step process of the anodic oxidation of APO. No reduction peaks were observed, when the polarization direction was reversed to cathodic, even when *E*_λ_ was close to the oxidation peaks. The products of both steps of the anodic oxidation of APO are unstable and undergo irreversible homogeneous chemical reactions, resulting in final products that cannot be reduced within the available potential range. The course of the CV curves allows the voltammetric analysis of only the first peak. The course of the CV curves allows the voltammetric analysis of only the first peak. Figure 5B shows that, at the APO oxidation peak current, *I*_pa_ increases as the scan rate, *v*, increases. According to the Randles–Sevcik equation, the linearity of the *I*_pa_ vs. *v*^1/2^ plot (Figure 5C) indicates that the first step in APO oxidation is diffusion-controlled. At higher scan rates, the deviation from linearity may be attributed to an increase in the degree of irreversibility of the electron exchange reaction [25]. Figure 5B shows a slight shift of the peak potentials toward more positive values as *v* increases, indicating the quasi-reversibility of the first step of the anodic oxidation of APO. This conclusion is supported by the width of the anodic peaks (*E*_pa_–*E*_pa/2_), where *E*_pa_ and *E*_pa/2_ represent the anodic peak potential and the potential at the half the height of the peak, respectively. The values obtained (0.055–0.063 V in the range of *v* from 6.2 to 50 mV s^−1^) differ slightly from those expected for a reversible one-electron transfer (0.0565 V [24]).

**Scheme 1:**
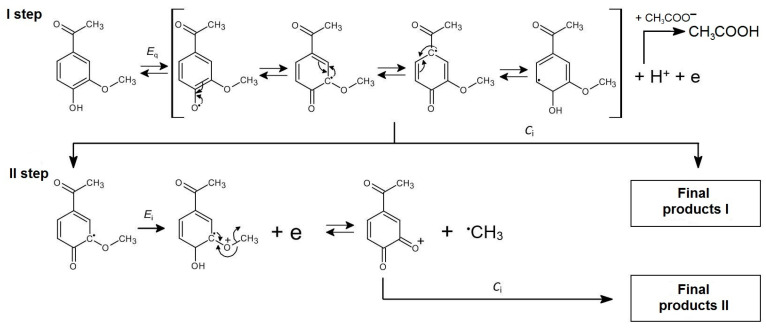
Proposed mechanism of APO oxidation.

Based on the study results, a predicted two-step mechanism for the anodic oxidation of APO was proposed (Scheme 1). The APO radical is the product of the first step of the electrode reaction, which involves a quasireversible (*E*_q_) and diffusion-controlled exchange of one electron and one proton. The presence of acetate anions in the solutions facilitate the process by binding protons and shifting the equilibrium toward the products. Consequently, a decrease in the potentials of the APO oxidation is observed (Figure 4). Both the APO radical and the APO cation (the product of the irreversible exchange reaction of one electron in the second step (*E*_i_)), are unstable and undergo subsequent irreversible chemical reactions (*C*_i_). It is also likely that, in the second step of electron exchange, an unstable methyl radical may be formed instead of the APO cation. The final products I and II of the anodic oxidation of apocynin are not reduced within the available potential range. The absence of reduction peaks on the CV curves (Figure 5) demonstrates this. The proposed mechanism of anodic oxidation of APO can therefore be described as *E*_q_*C*_i_*E*_i_*C*_i_. It is worth emphasizing that the presented mechanism is only probable. Our laboratory was unable to identify the primary and final products formed during the anodic oxidation of apocynin.

### 3.3. Analytical Characteristics

**Figure 6:**
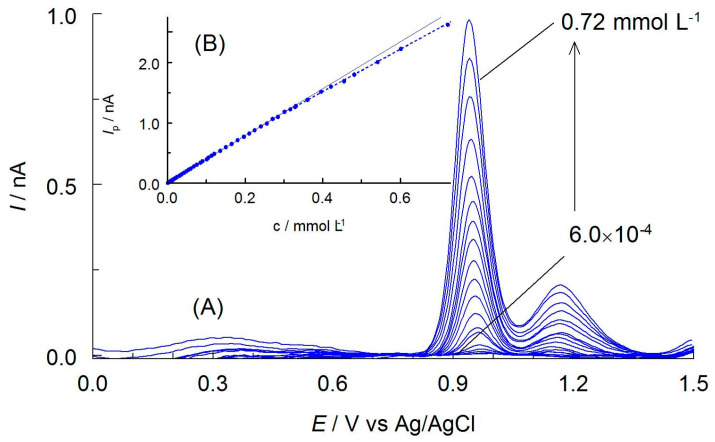
(**A**) DPV curves recorded on CF microelectrode containing different concentrations of APO ranging from 6.0 × 10^−4^ to 0.72 mmol L^−1^. Other components of the solutions are as shown in Figure 3. (**B**) Calibration plot for APO.

The first stage of analytical investigation was to construct a calibration curve and determine analytical parameters. For this purpose, DPV curves were recorded for the different APO concentrations under optimized environmental conditions and using optimal DPV parameters (Figure 6A). The selected analytical signal was the peak at a potential of 0.925 V. It was observed that this peak current increased linearly with increasing apocynin concentration in solutions in the range of 2.7 × 10^−6^–2.6 × 10^−4^ mol L^−1^ (Figure 6B). The calibration curve was described by the equation: *I*_p_ (nA) = (4.068 ± 0.0316) × *c* (mmol L^−1^) + (0.00089 ± 0.0011) with correlation coefficient, *r* = 0.9999. The limit of detection (*LOD*) of APO was calculated from the standard deviation of the intercept, *S*_b_ and the slope of the calibration curve, *a* (*LOD* = 3.3*S*_b_/*a* [26,27]), and was determined at 8.9 × 10^−7^ mol L^−1^. The limit of quantification (*LOQ* = 3*LOD*) was estimated at 2.7 × 10^−6^ mol L^−1^.

Table 1 presents the comparison of the main analytical parameters for the developed voltammetric method of apocynin determination with the literature data from various chromatographic procedures. The proposed method is characterized by comparable linearity range and the detection limit. The exceptions are the values obtained with LC–MS/MS. These parameters confirm the high sensitivity and utility of the developed method. Therefore, it can be an effective alternative to the prevailing chromatographic methods for APO determination. The small sample amount, minimal solvent/reagent consumption and low waste generation make this method particularly attractive and compliant with the principles of “green analytical chemistry” [28].

A series of repetitive electrochemical measurements (n = 10) were performed to test the precision of the voltammetric method. The intra-day repeatability of the peak currents for solutions of 3.0 × 10^−6^, 6.0 × 10^−5^, and 2.4 × 10^−4^ mol L^−1^ were excellent, and the relative standard deviations, *RSD*, were not greater than 2.4, 0.4 and 0.2%, respectively. The inter-day repeatability of the results was determined by measuring the peak currents for five days using the same solutions. The *RSD* values obtained did not exceed 3.0, 0.7, and 0.5%, respectively. The peak potential characteristic for APO was stable in the linear ranges of the calibration curve.

### 3.4. Selectivity

**Figure 7:**
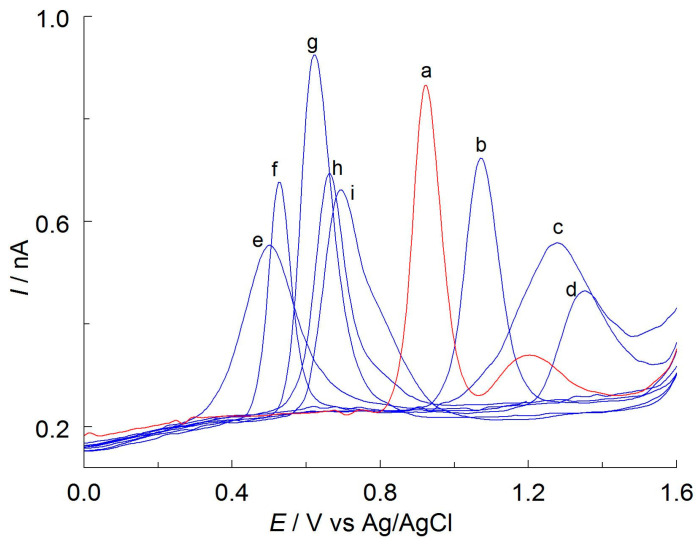
Selected DPV curves recorded on a CF microelectrode in solutions containing APO and possible interfering species (all at the concentration 0.25 mmol L^−1^): (a) APO, (b) α-lipoic acid, (c) BHT, (d) α-TOAc, (e) TBHQ, (f) α-tocopherol, (g) protocatechuic acid, (h) δ-tocopherol, (i) BHA. Other components of the solutions are as shown in Figure 3.

To evaluate the selectivity of the developed procedure, the effects of possible interfering species were investigated. The tests were carried out for butylated hydroxyanisole (BHA), butylated hydroxytoluene (BHT), tert-butylhydroquinone (TBHQ), α-lipoic acid, α-tocopherol, δ-tocopherol, α-tocopheryl acetate (α-TOAc), chlorogenic, caffeic, rosmarinic and protocatechuic acid, and also inorganic ions Na^+^, K^+^, Ca^2+^, Mg^2+^, Cl^−^, NO_3_^−^, and SO_4_^2−^. These compounds can be found in herbal extracts and dietary supplements along with APO. Figure 7 shows the selected DPV curves of potential interfering species. As can be seen, none of these signals overlap with the anodic oxidation curve of APO. The effect of the 10-fold excess of the interferents concentration on the peak currents of apocynin did not exceed 4%. The presented voltammetric method can therefore be considered specific.

### 3.5. Extraction

**Figure 8:**
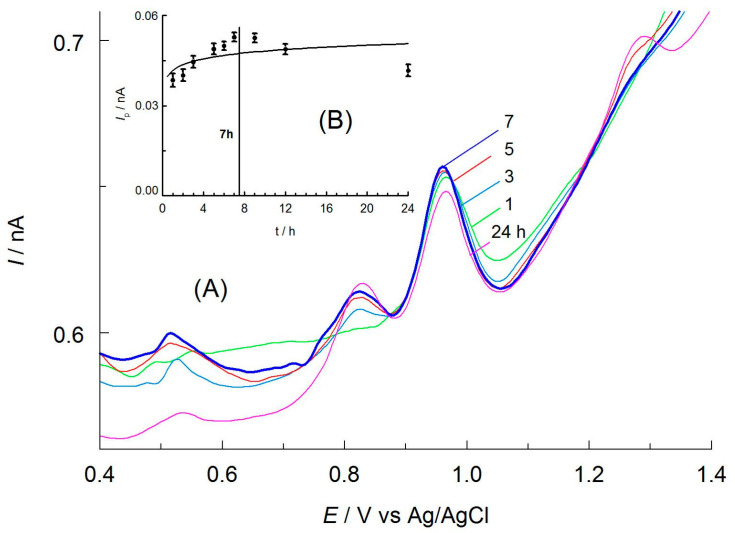
(**A**) Selected DPV curves of APO oxidation recorded on CF microelectrode in Kutki extracts spiked with apocynin (100 μL extract/25 mL) after various times of extraction. Other components of the solutions are as shown in Figure 3. (**B**) Influence of the extraction time on the DPV peak current (*I*_p_) of the APO oxidation.

Preliminary investigations showed that acetonitrile was the most effective solvent for the extraction of apocynin from real samples among the extractants tested (methanol, ethanol, acetonitrile, and hot water). The curves recorded in the solutions containing AN extract were well shaped, symmetrical, and reproducible. The study’s subsequent stage was to establish the optimal time of the extraction process, allowing complete separation of the analyte from the plant matrix. For this purpose, a known amount of apocynin (5 mg) was added to a sample of the Kutki dietary supplement (1 g) and 10 mL acetonitrile. They were then mechanically shaken. After that, an appropriate volume of the filtered extract (100 μL) was diluted in a 25 mL volumetric flask with an experimentally chosen medium. DPV curves were recorded in solutions containing extracts taken after 1 to 24 h. A gradual increase in the peak current was observed, caused by an increase in apocynin concentration in the extracts over time (Figure 8). This increase was greatest for extraction times of up to 7 h. After this time, the peak current gradually stabilized. This means that the concentration of APO in the extract reached the maximum as a result of its content in the sample. It was concluded, based on the results obtained, that the optimal extraction time for apocynin from plant materials should not be less than 7 h.

### 3.6. Recovery Studies

The reliability of the method was verified by performing control determinations at three concentration levels. For this purpose, a plant that does not contain apocynin, i.e., rosemary, was used as a matrix. Specified amounts of APO were added to rosemary in the samples, and they were then extracted with 10 mL of acetonitrile as described in Section 2.3. Next, appropriate volumes of extracts were diluted with an experimentally chosen medium to obtain apocynin concentrations of 1.20 × 10^−5^, 3.01 × 10^−5,^ and 6.02 × 10^−5^ mol L^−1^. An amount of 2.0 mL of these solutions were transferred to an electrochemical cell, and DPV curves were recorded before and after the addition of the APO standard solution in 50 μL portions. The concentrations of the standard solutions were 1.68 × 10^−4^ (control 1 and 2) and 9.19 × 10^−4^ mol L^−1^ (control 3). The determinations were repeated five times. The concentrations of APO were converted per 1 g of the dry sample and were statistically examined (Table 2). The average apocynin content in the control solutions was found to be very close to the introduced amounts (*R* = 99.9–100.1%). This indicates that the developed method is accurate, with a high level of precision as the *RSD* value does not exceed 0.6%.

### 3.7. Real Sample Analysis

**Figure 9:**
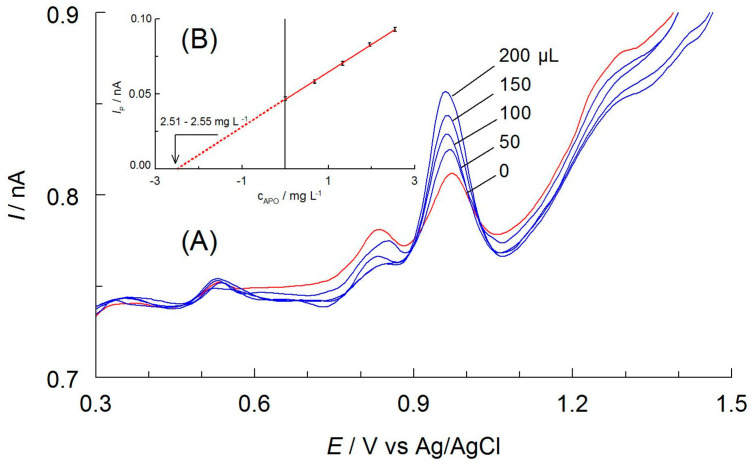
(**A**) DPV curves recorded on CF microelectrode in Kutki extract prepared with acetonitrile spiked with apocynin (2.01 mg L^−1^ APO) and after additions of APO standard solution (*c* = 1.68 × 10^−4^ mol L^−1^ (27.96 mg L^−1^), the volumes in μL are given at curves). Other components of the solutions are as shown in Figure 3. (**B**) Calibration curve in the standard addition method.

Positive test results enabled the determination of APO in the dietary supplement. The analytical procedure was the same as for the control determinations. There was a well-defined oxidation peak at about 0.9 V on the DPV curves of the Kutki extracts, indicating the oxidation of apocynin. This was confirmed using the standard addition method (Figure 9). The experimental results presented in Table 2 confirm the very good precision of the developed method as the *RSD* values did not exceed 0.9%. Based on the data obtained from control determinations, it can be concluded that the results are reliable.

## 4. Conclusions

In this study, the first voltammetric method for the sensitive and selective determination of apocynin was proposed, using a carbon fiber microelectrode (33 μm in diameter) as the sensor. Due to limited solubility of APO in water, a mixture of anhydrous acetic acid containing 20% *v*/*v* acetonitrile and 0.1 mol L^−1^ sodium acetate was used as the environment. The developed method is based on a detailed examination of the electrochemical properties of a CF microelectrode using LSV and DPV techniques, and CV on a GC electrode. It was found that APO undergoes two-step anodic oxidation in acetic acid, which involves the exchange of two electrons and one proton. The APO radical and APO cation, which are the probable products of the first and second steps of the electrode reaction, respectively, are unstable and undergo subsequent chemical transformations near the surface of the working electrode. Therefore, the proposed mechanism of this process can be described as *E*_q_*C*_i_*E*_i_*C*_i_. The developed voltammetric method for APO determination is based on the signal at a potential of 0.925 V vs. Ag/AgCl, which corresponds to the first step in the anodic oxidation of the analyte. The DPV procedure showed a good linear response in the concentration range of 2.7 × 10^−6^–2.6 × 10^−4^ mol L^−1^. The calculated *LOD* and *LOQ* values were 8.9 × 10^−7^ and 2.7 × 10^−6^ mol L^−1^, respectively. The utility of this method was successfully demonstrated in the determination of APO in herbal extracts and dietary supplements.

In summary, the method developed for determining APO based on CF as a microelectrode material in an acetic acid environment has a wide linear range, low detection and quantification limits, satisfactory reproducibility, selectivity, sensitivity, and precision. Additionally, while meeting the principles of green chemistry, it is safe for both humans and the environment. It can be an effective analytical tool for applications in herbal, food, and pharmaceutical analysis.

## Figures and Tables

**Table 1:** Comparison of analytical parameters for analysis of APO obtained with different techniques.

Method	Application	Linear Range (mol L^−1^)	*LOD* (mol L^−1^)	*LOQ* (mol L^−1^)	Ref.
HPLC-UV	rat plasma	1.2 × 10^−6^–6.0 × 10^−4^		1.2 × 10^−6^	[14]
HPLC-UV	bovine serum albumine	3.0 × 10^−5^–6.0 × 10^−4^	4.7 × 10^−7^	1.4 × 10^−6^	[15]
LC-MS/MS	rat and human plasma	6.0 × 10^−9^–6.0 × 10^−6^		6.0 × 10^−9^	[16]
DPV(CF)	herbal extracts	2.7 × 10^−6^–2.6 × 10^−4^	8.9 × 10^−7^	2.7 × 10^−6^	This work

**Table 2:** Results of the apocynin determination in control solution and dietary supplement by DPV.

Sample	APO Taken/ mg g^−1^	^(1)^ APO Found/ mg g^−1^	^(2)^ *RSD/*% (n = 5)	^(3)^ *R*/%
Control 1 (rosemary matrix)	4.97	4.97 ± 0.03	0.6	100.0
Control 2 (rosemary matrix)	12.39	12.38 ± 0.04	0.4	99.9
Control 3 (rosemary matrix)	24.36	24.38 ± 0.07	0.3	100.1
Kutki	-	1.27 ± 0.02	0.9	-
Kutki with APO added	4.92	6.21 ± 0.04	0.5	126.2

^(1)^ x = x_av_ ± t_0.95_ S_av_ for n = 5 and *t*_0.95_ = 2.776 (tabulated), S_av_ denote standard deviation of mean, ^(2)^ relative standard deviation, ^(3)^ Recovery, *R* = (x_av_/amount involved) × 100%.

## Data Availability

Data are contained within the article.
